# Neuroautonomic Adaptive Resilience (NAR): The Proposal of a New Mechanism in the Context of the Neuroscience of Cognitive and Affective Aging. Definition and Operationalization

**DOI:** 10.1002/wcs.70038

**Published:** 2026-08-11

**Authors:** Giulia Fusi, Barbara Colombo, Simona C. S. Caravita

**Affiliations:** ^1^ Department of Human and Social Sciences University of Bergamo Bergamo Italy; ^2^ School of Psychology Fielding Graduate University Santa Barbara California USA; ^3^ Università “Mercatorum” di Roma Rome Italy

## Abstract

Resilience mechanisms to counteract aging and the onset of neurological and neurodegenerative diseases are becoming an increasingly central issue due to the aging of the population. Several mechanisms have been hypothesized, and in the context of cognitive resilience, the construct of cognitive reserve has attracted considerable interest. However, it is becoming increasingly clear that there are several possible mechanisms of resilience (e.g., motor, affective, behavioral, etc.) which interact with each other, influencing inter‐individual variability in healthy and pathological aging trajectories. However, to date, central‐peripheral processes have not yet been explicitly incorporated as a resilience mechanism in models of cognitive and affective aging. This perspective article therefore aims to propose a new resilience mechanism, the “Neuroautonomic Adaptive Resilience” (NAR) that implicate a flexible and efficient integration of the central and the autonomic nervous system, allowing individuals to adapt effectively to stressors throughout their lives. This perspective proposes its theoretical framework, its operationalization, and its integration with existing concepts in the field of cognitive and affective neuroscience of aging.

## Graphical abstract

Understanding resilience mechanisms in aging and identifying early non‐invasive biomarkers is crucial. This perspective article proposes the Neuroautonomic Adaptive Resilience (NAR)—a cross‐system balance between autonomic and central nervous systems—as a mechanism that can index how individuals can adapt flexibly and efficiently to stressors. This mechanism, interacting with other already hypothesized mechanisms and reserve processes described in aging neuroscience research, might protect individuals from pro‐inflammatory processes, resulting in better cognitive and mental health outcomes across the lifespan.

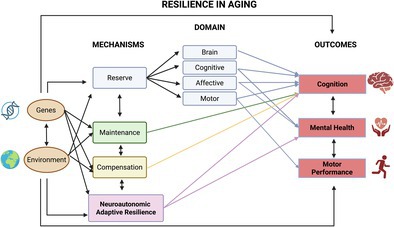

## 1 Headline

In the last years, many concepts related to cognitive reserve and resilience have been theorized to explain the non‐linear relationship between brain damage and cognitive and behavioral manifestations both in aging and in different neurological conditions. Several constructs have been theorized, such as affective (Di Rosa 2024), motor (Giustiniani and Quartarone 2024) or behavioral (Premi et al. 2013; Guan et al. 2024) reserve, however sometimes modest exploration of a concrete operationalization of these new constructs has been proposed. Moreover, a call for the identification of resilience biomarkers across the lifespan has been put forward (Arenaza‐Urquijo and Vemuri 2020).

Here, we propose a new mechanism, named “Neuroautonomic Adaptive Resilience” (NAR), that can be integrated in the panorama of cognitive and affective resilience within a lifespan perspective, proposing its theoretic definition and operationalization.

## 2 Introduction

Aging is a complex and multidimensional process that affects neurobiological and psychological functions at multiple levels (Cabeza et al. 2018). In the field of cognitive neuroscience, it is well known that both structural and functional changes occur in the brain throughout the lifespan (see Lee and Kim 2022 for a review) leading to changes in cognitive performances (see e.g., Brito et al. 2023; Salthouse 2004, 2010; Spreng and Turner 2019). However, there is a high degree of inter‐individuality in the trajectories of cognitive aging (Cabeza et al. 2018) and that this may be due to the impact of multiple risk and protective factors (see for example Livingston et al. 2024; Reuter‐Lorenz and Park 2014).

One of the most interesting risk factors is the negative effect of chronic stress (Livingston et al. 2024). Stress—that can be described as a natural and adaptive human response that allows individuals to efficiently address life challenges and threats (World Health Organization 2023)—is necessary for adaptation and homeostasis. However, sometimes, if it turns into chronic or badly managed stress with prolonged physiological dysregulation (i.e., exaggerated or blunted stress responses) can have harmful effects both on brain and overall body health (Thayer et al. 2021), so much that is considered a common risk factor (75%–90%) of most chronic, noncommunicable diseases (Agorastos and Chrousos 2022). Moreover, in a lifespan perspective, stress is considered a factor that can accelerate all the physiological processes linked to biological aging (Lavretsky and Newhouse 2012; Polsky et al. 2022; Thayer et al. 2021). One of the main hypothesized mechanisms for this acceleration is linked to the increase of pro‐inflammation processes (i.e., “inflammaging,” Franceschi et al. 2000). Inflammaging—whose biological mechanisms are beyond the scope of this article but can be explored in greater depth for example in Giunta et al. (2024) or Olivieri et al. (2024)—is considered a key factor that can impact aging trajectories and also exacerbate the pathogenesis of some neurodegenerative disorder such as Alzheimer Disease (Kinney et al. 2018; Lohman et al. 2025).

In this context, recently, research has evidenced how specific mechanism linked to the imbalance of Autonomic Nervous system (ANS), and in particular the Sympathetic Nervous System (SNS) overflow reported during aging, could contribute to fuel inflammaging (Giunta et al. 2024). In this sense, since ANS can regulate inflammation both in chronic and acute conditions, autonomic dysfunction may affect the risk for mood and cognitive disturbance in older individuals (Lavretsky and Newhouse 2012) and can have a pivotal influence on the onset and progression of many age‐related diseases (Lohman et al. 2025). ANS balance can therefore represent a protective factor that can be well integrated in the literature on cognitive and affective resilience. However, many existing models of aging resilience explain the variability of cognitive trajectories emphasizing brain or cognitive mechanisms, while giving limited attention to dynamic central–peripheral regulatory processes that may shape adaptation across the lifespan.

The following paragraphs aim to present the theoretical assumptions of our hypothesis in detail, providing a clear perspective on the operationalization of the Neuroautonomic Adaptive Resilience (NAR) construct and its positioning in the resilience framework, with particular emphasis on addressing the limited integration of central–peripheral regulatory mechanisms in current models of cognitive and affective aging.

## 3 Theoretical Background

### 3.1 Neurovisceral Integration Model: Heart Rate Variability as an Index of Top‐Down Control Signal

Our proposal takes place in the light of the Model of Neurovisceral Integration (Thayer and Lane 2000; Thayer et al. 2009), which postulates a dynamic neural system that, through the flexible integration between emotional, attentional and autonomic systems, represents an interface between cognition and emotion (Park and Thayer 2014) allowing individuals for efficient emotional self‐regulation (Thayer and Lane 2000) and for flexible goal‐directed behavior (Cacioppo et al. 2007; Thayer and Lane 2000). This complex integration between the Central Nervous System (CNS) and ANS is allowed through direct and indirect pathways (both excitatory and inhibitory) within frontal‐subcortical areas that can be resumed in the “Central Autonomic Network” (CAN) (for a detailed description of the brain area involved see Benarroch (1993) or Lamotte et al. (2021)). CAN is a complex network that allows internal regulation through brain‐heart and cognition‐emotion interaction (Benarroch 1993). It plays a critical role in the stress response allowing the organism to respond to demands from the environment (Lamotte et al. 2021) and to organize behavior effectively (Thayer and Lane 2000).

The interplay of sympathetic and parasympathetic (vagal) outputs of the CAN on the sino‐atrial node of the heart is indeed considered the source of the complex beat‐to‐beat variability (or Heart Rate Variability, HRV) that is characteristic of a healthy and adaptive organism (Thayer and Lane 2000) and that is responsive to both acute and chronic stress (see Brugnera et al. 2017, 2018; Kim et al. 2018). Thus, HRV is nowadays considered as an index of the central–peripheral neural feedback and of CNS–ANS integration (Thayer and Lane 2000) but also an index of inflammatory processes (Williams et al. 2019). Moreover, other authors evidenced that this index can be also considered as a peripheral marker of prefrontal inhibitory control (i.e., top‐down signal) over emotional stress (Appelhans and Luecken 2006) and over prolonged peripheral stress responses (Thayer et al. 2021). Or again, a proxy for “vertical integration” of the brain mechanisms with peripheral physiology that guide flexible control over behavior (Thayer et al. 2012) and a psychophysiological marker for environmental adaptation (Visnovcova et al. 2013).

According to this, individual differences in HRV have been related to performance on tasks associated with executive functioning (Forte et al. 2019; Forte and Casagrande 2025; Thayer et al. 2009; Perna et al. 2020), emotional regulation abilities (Balzarotti et al. 2017; Nasso et al. 2019) which imply prefrontal cortical activity (Thayer et al. 2009) and cognitive reserve (Forte and Casagrande 2025). Several research, in line with these findings, have evidenced that reduced HRV—usually due to decreased parasympathetic markers and a shift to sympathetic dominance—is generally associated with poorer emotional adaptability, increased psychopathology risk (see e.g., Alvares et al. 2016; Chalmers et al. 2014; Faurholt‐Jepsen et al. 2017; Koch et al. 2019) and last but not least, cognitive decline during the lifespan (Jandackova et al. 2024).

Perna et al. (2020) have proposed the idea that HRV may be considered a global index of an individual's flexibility and capacity to adapt to stress. The authors suggest that vagally mediated indexes of HRV may be plausible, non‐invasive, and easily applicable transdiagnostic biomarkers of a generic “mental health resilience” that reflects the efficiency of the interplay of multiple systems—physiological, emotional, and cognitive—in the responses to stressors.

However, in this theory, only vagally mediated indexes are contemplated, thus considering only one side of the ANS activity. Moreover, this theory is not grounded in the cognitive aging panorama. This highlights the need for a resilience framework that explicitly models the dynamic integration of sympathetic and parasympathetic activity within a lifespan and aging perspective.

### 3.2 ANS and Stress: The Cross‐System Autonomic Balance (CSAB)

As presented in the previous paragraph, HRV is mainly used as a parasympathetic index of vagal withdrawal on the heart. However, this is only one aspect of what happens when individuals are confronted with a stressor. A dynamic balance of ANS is required in the different phases of stress response (i.e., anticipation, confrontation and recovery; see Flores‐Kanter et al. 2021). In particular, during the phase of confrontation a parasympathetic activity decline (vagal withdrawal) with reciprocal increase of sympathetic activity (arousal) occurs (Flores‐Kanter et al. 2021). If HRV indexed the activity of the parasympathetic nervous system, electrodermal activity (EDA) instead provides complementary and clear information about sympathetic excitation (Visnovcova et al. 2013). These two non‐invasive indices of sympathovagal balance in response to stress (Visnovcova et al. 2013, 2016) can be therefore integrated as a cross‐system autonomic balance index providing a more comprehensive and robust characterization of ANS functioning and balance (Ghiasi et al. 2020; Stone et al. 2020).

Following what was already described in the previous paragraphs, a proper functioning of the sympathetic‐parasympathetic dynamic balance at rest, as well as in all the phases of the response to stress, enables adaptive response to load—allowing for better allostasis (Visnovcova et al. 2013). In line with this prediction, even if the literature in this area is scarcer, results seem to be in line with what is presented in the last paragraph. Patients (both psychiatric and neurologic) with an impaired or absent EDA bottom‐up signaling showed worse cognitive performances, again mainly in the executive domain (Bechara et al. 2000; Nilsson et al. 2015; Pruneti et al. 2023) and higher risk of affective alterations such as depressive symptoms (see Sarchiapone et al. 2018 for a review). Moreover, more in general, lower cross‐system autonomic balance—indicating sympathetic dominance—has been associated with different affective disorders such as higher depressive and anxiety symptoms (Stone et al. 2020).

Further studies are needed in this area, but these studies seem to point again to the importance of how bottom‐up and top‐down signals from CNS and ANS interact and are flexibly integrated during all the diverse phases of stress allowing individuals to adapt efficiently to cognitive and/or emotional challenges (Flores‐Kanter et al. 2021).

## 4 Cross‐System Autonomic (Im)Balance in the Context Aging: The Proposal of the Neuroautonomic Adaptive Resilience (NAR) Mechanism

During the aging process there is a natural change in ANS functioning: on the one hand a blunted reactivity has been found (Giunta et al. 2024); on the other hand, an imbalance toward a relatively greater sympathetic activation that is driven by decreased parasympathetic activity has been evidenced by several studies (Giunta et al. 2024; Olivieri et al. 2024; Thayer et al. 2021). This ANS imbalance—via an impaired vagal regulation of inflammatory response—contributes to the pro‐inflammatory state that fuels the previously cited inflammaging (Giunta et al. 2024) and has a significant role in the incidence of multiple age‐related conditions ranging from cardiovascular to neurodegenerative disorders (Butterfield 2002; Lohman et al. 2025; Schwarz et al. 2024).

The relationship between stress, ANS imbalance and inflammaging, although already present in the literature (see Perna et al. 2020), has never been conceptually linked to the constructs of resilience that are pivotal in the context of the cognitive neuroscience of aging. Before introducing the NAR construct a brief paragraph is included to provide the main definitions and operationalizations of constructs and mechanisms already proposed in the relevant scientific literature of resilience in aging—such as brain, cognitive, motor, affective and behavioral reserve—in order to better highlight the overlaps and differences among the proposed constructs.

### 4.1 Resilience in Aging: An Overview

As anticipated in the previous paragraphs, the rapid aging of the global population and the resulting increase in the incidence of neurological and neurodegenerative disorders have attracted significant interest from researchers seeking to identify protective and modifiable factors throughout the lifespan. Generically, we will refer to this area of studies, as concerning “resilience,” a term that subsumes any concept that relates to the capacity of the brain to maintain cognition and function (Stern et al. 2023) in the face of both healthy and pathological aging processes or a broader concept that is described as “the ability to recover from or adjust easily to misfortune or change” and that can be applied to different areas such as brain, cognition and aging but also psychological constructs (Kremen et al. 2022). Thus, the concept was then extended to other areas as well, such as the emotional, motor, and behavioral domains: in general, the mechanisms hypothesized to date share the common feature of positing a “reserve” mechanism that results in “better‐than‐expected outcomes” (e.g., cognitive reserve has been hypothesized to explain better‐than‐expected cognition, motor reserve to explain better‐than‐expected motor performance, and so on). The purpose of this article is not to present, in a systematic and in‐depth manner, all the constructs proposed in the literature regarding resilience, but rather to describe the main mechanisms that have been hypothesized and/or tested in recent decades to specifically and thoroughly situate the NAR mechanism within this context. For this reason, these concepts are summarized and presented in Table 1.

**TABLE 1:** Description of the main constructs and mechanisms proposed in the context of resilience in healthy and pathological aging with identified proxies, proved or hypothesized outcomes and limitations.

Construct	Definition	Specific mechanisms	Proxy	Outcomes	Limitations
BRAIN RESERVE (BR)	BR has been used to reflect the neurobiological status of the brain (numbers of neurons, synapses, etc.), or the total neural resources/neurobiological capital (Arenaza‐Urquijo and Vemuri 2020) at any point in time (Stern et al. 2023).	BR does not involve active adaptation of functional cognitive processes in the presence of injury or disease as does CR (Stern et al. 2023).	Number of neurons Number of synapses	Cognitive	Passive
COGNITIVE RESERVE (CR)	CR is the ability to optimize or maximize performance through the differential recruitment of brain networks, which may reflect the use of alternative cognitive strategies and is described as a property of the brain that allows for cognitive performance that is “better than expected” given the degree of life‐course related brain changes and brain injury or disease (Stern 2002; Stern et al. 2023).	Reserve is about augmenting resources beyond their current level, cumulatively influencing neural capacity and neural efficiency (Cabeza et al. 2018).	Educational level Work Leisure activities Bilingualism Divergent thinking (etc.)	Cognitive Psychological	Not comprehensive Relatively static
MANTEINANCE	Refers to the preservation of neural resources referring to an ongoing repair and replenishment of the brain in response to damage incurred at the cellular and molecular levels (Cabeza et al. 2018).	Maintenance is about returning resources to their former higher level. Influence neural mechanisms of repair and plasticity (and others not yet identified) and can relate to different aspects of the brain (gray and white matter), to different neurotransmitter systems or even to different brain regions. Specific mechanisms remain to be determined but likely include both neural (e.g., neurogenesis) and non‐neural (e.g., vascular changes) components.	—	Cognitive	Require longitudinal assessment Not specified proxy/measurements
COMPENSATION	Compensation should be used to refer to the cognition‐enhancing recruitment of neural resources in response to relatively high cognitive demand (Cabeza et al. 2018).	*Selection*: Occurs when older adults engage in a process not currently recruited by young adults but available to young adults, who may use it in other tasks or conditions *Upregulation*: Occurs when neural activity increases in response to greater task demands *Reorganization*: Occurs when older adults may use a neural mechanism to respond to aging‐induced losses that is not available to younger individuals.	Comparison in functional activations (younger vs. older adults)	Cognitive	Require complex experimental paradigms and activation measurements (not always available)
AFFECTIVE RESERVE (AR)	“*Affective Reserve can be defined as the capacity to regulate affective states and dispositions (*i.e., *emotions, mood, motivation) that helps to explain the differential susceptibility and response to stressful and adverse life events, pathologies, and aging*” (Di Rosa 2024, pg. 1). It includes the concepts of both emotional and motivational reserve but going beyond them.	Emotional Reserve to refer to the adaptability of the individual emotional functioning, which helps to explain individual differences in affective functioning (Pinto et al. 2023). Motivational reserve can be defined as a set of motivational abilities or processes that provide the individual with resilience to neuropathological deterioration (Forstmeier and Maercker 2008).	Attachment style Emotional regulation Motivation regulation Coping behaviors Social support	Affective Psychological Cognitive	Lack of clear measurement proposal
MOTOR SYSTEM RESERVE (MR)	Moto system reserve is a comprehensive model of plasticity‐based reserve in the motor system that includes different mechanisms (Giustiniani and Quartarone 2024).	*Motor reserve* (MR): Active process explaining the discrepancy between the severity of motor symptoms exhibited by patients with Parkinson's disease (PD) and their levels of brain degeneration *Cerebellar reserve*: Conceptualized as the capacity of the cerebellum to compensate and restore functions in case of motor damage, tissue damage, or loss of functioning *Muscular unit reserve*: Identified in patients with spinal muscular atrophy (SMA) in terms of an unexpected increase in the amplitude of EMG activity immediately before failure and reflecting the recruitment of new motor units (reserve).	Physical activity	Motor/physical Cognitive	Not exhaustive definitions or clear measurement methods
BEHAVIORAL RESERVE	Data on FTD patients suggest that reserve mechanisms act in a multi‐modal way, not only cognitive performances might be influenced by reserve mechanisms but can potentially be applicable also to different types of neuropsychiatric symptoms (Premi et al. 2013).	Not specific for behavioral reserve	Residual approach between observed and expected behavioral alterations	Behavioral (mainly studied in FTD patients)	Different name linked to outcome for the same underling mechanism (reserve)

From this overview, it seems clear that aging trajectories are influenced by multiple mechanisms that are not mutually exclusive but interact to determine these trajectories and, consequently, interindividual variability. However, most of the empirical work presented here has focused attention on purely cerebral (brain reserve, BR; Stern 2002, 2009), cognitive (Stern 2009; Pappalettera et al. 2024; etc.), neural (Stern 2006) and/or affective mechanisms (Di Rosa 2024) giving limited attention to dynamic central–peripheral regulatory process that may shape adaptation across the lifespan. None of these studies have indeed considered the possible role of an efficient interaction and integration between CNS and ANS peripheral signals as a central resilience mechanism in aging even if several studies have proved how these mechanisms: are intrinsically interconnected; are essential to human ability to flexibly control over complex behavior (see Mather and Thayer 2018; Thayer et al. 2012) and therefore, in this context, to easily adapt to the changes imposed by aging and pathology (Thayer et al. 2021); provide a protection both for cognitive and mental (affective/psychological) health (Perna et al. 2020).

### 4.2 The NAR Mechanism Proposal

Thus, we propose another mechanism that can be integrated in the panorama of resilience in aging: the “Neuroautonomic Adaptive Resilience” (NAR) mechanism. NAR, indeed, can be described as a resilience mechanism characterized by flexible and dynamic coordination between central regulatory influences and autonomic responses—operationalized through complementary indices of vagal regulation (HRV) and sympathetic activation (EDA) that allows individuals to self‐regulate and to adapt efficiently to cognitive and emotional challenges along all the lifespan. In this sense, NAR is conceptualized as a new resilience mechanism emphasizing real‐time adaptive self‐regulation rather than as a reserve construct based on accumulated or proxy‐based indicators.

In particular, in line with what is presented in the previous paragraphs, higher NAR would be represented by the ability of the individuals to flexibly maintain a cross‐system autonomic balance through all the phases of the stress response (baseline, confrontation and recovery), allowing them to efficiently adapt their behavior to internal and external stressors. More in detail, higher NAR would be related to (i) good vagal regulation at baseline that corresponds to higher basal HRV and an efficient integration of anticipatory signals from EDA, both linked to better emotional regulation and executive functioning (Balzarotti et al. 2017; Forte et al. 2019) and essential for self‐regulatory systems (Colombi et al. 2026); (ii) an adequate vagal withdrawal and sympathetic mobilization during the challenge with a balanced decrease in HRV and increase in EDA compared to baseline; and (iii) an efficient recovery with the indices returning to their baseline levels.

Operationally, NAR may be quantified either through composite indices integrating HRV and EDA measures (e.g., Ghiasi et al. 2020) or through autonomic response profiles across stress phases, depending on research and clinical aims. All these mechanisms seem to be grounded in the activity of the prefrontal functioning, fundamental for emotional regulation, executive functioning and more generally goal‐directed behavior, that through the complex cortical–subcortical pathways of the CAN, represent the potential neural substrates of NAR. However, neural pathways are beyond the scope of this article and must be explored, studied, and tested in subsequent articles.

In the long term, ANS balance allowed by the NAR mechanism might protect from the pro‐inflammatory processing which is usually triggered by an imbalance with sympathetic dominance that occurs during aging, making NAR the perfect candidate for preventive intervention to sustain healthy and successful aging. Therefore, this mechanism would make individuals more resilient to stressors but also to the different biological changes that occur along the lifespan enabling individuals to maintain good mental but also cognitive health. For a resume of the operationalization of NAR see Table 2.

**TABLE 2:** Resuming the operationalization of NAR construct: Definition, mechanisms, measurement, expected outcomes.

Definition	General mechanism	Biological mechanism	Measurement	Expected outcomes	Hypothesized validation protocol
NAR is a resilience mechanism consisting of a flexible and dynamic interaction between autonomic and central nervous systems that allows individuals to self‐regulate and to adapt efficiently to cognitive and emotional challenges and to stressors along all the lifespan sustaining the individual to maintain cognitive and mental health in the face of aging and/or adverse brain‐related change.	Adaptive self‐regulation: autonomic sympathetic—parasympathetic balance.	Protect from pro‐inflammatory mechanisms due to chronic or badly managed stress.	Cross‐system autonomic balance (CSAB) assessed by HRV and EDA indices.	Better cognitive (especially executive) and affective (self‐regulation and general mental health) functioning.	NAR operationalized and assessed through CSAB measured during baseline, stress (cognitive or affective) confrontation and recovery should predict both cognitive and mental health outcomes beyond the effects of age, relevant clinical conditions, and established measures of cognitive and/or affective reserve (see also Figure 1 for a graphical representation).

*Note:* HRV and EDA are examples of indices of the possible individual differences in the autonomic response profiles across stress phases, and different validated measures may be used depending on study aims, protocol and target population.

**FIGURE 1:**
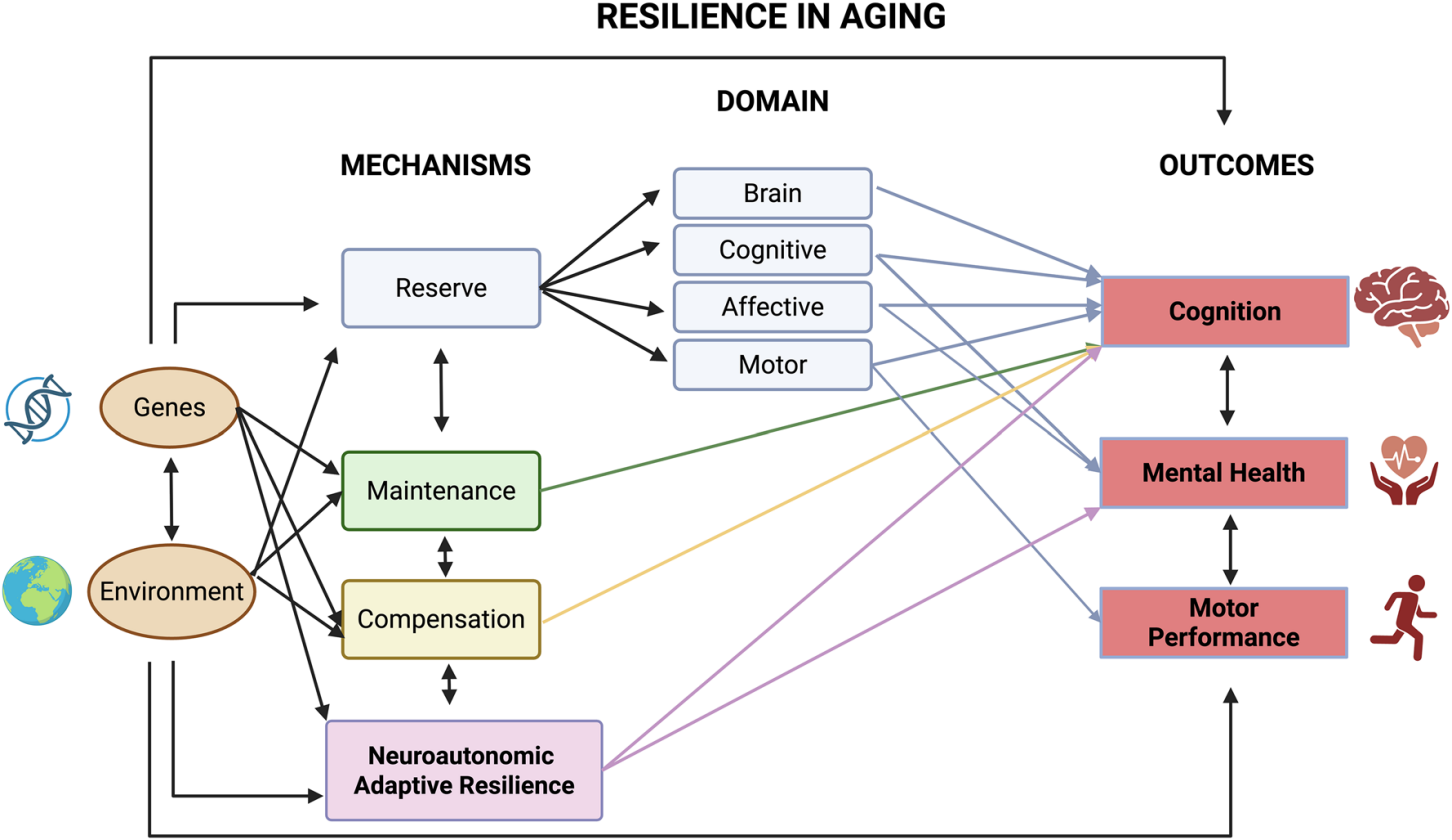
Graphical representation of the implementation of NAR mechanism in the context of resilience in aging and other constructs already hypothesized in this area of study. This is a schematic representation of NAR and mutual relationship with main hypothesized resilience mechanism in the field of cognitive and affective aging but does not represent a full representation of all the mechanisms in the literature.

Moreover, we propose that NAR can be considered a mechanism of resilience that can well be integrated in the panorama of resilience in aging, interacting with the other previously cited mechanisms potentially influencing positively both cognitive and mental health (see Figure 1 for a graphical representation).

To conclude, it is worth noting that NAR is built upon the neurovisceral integration model (see Section 3.1) but differs from it because it proposes the flexibility of central‐autonomic coordination as a mechanism of resilience across the lifespan, whereas the neurovisceral integration model describes the architecture and processes that support that regulation. Similarly, NAR differs from reserve, maintenance, and compensation because it concerns “real‐time” dynamic regulation, not accumulated resources, preservation of resources, or compensatory recruitment.

## 5 Implications, Advantages and Clinical Applications

The theorization of NARs can have several advantages. First of all, it is worth noting that, nowadays, cognitive reserve can be measured primarily using static questionnaires or proxies (see Nogueira et al. 2022 for a review of existing measures). This, with only a few attempts that have been put forward for more dynamic measures (Serra et al. 2017, 2019). On the contrary, the construct of NAR can be operationalized and assessed: (1) from direct but non‐invasive indexes; (2) dynamically, and (3) potentially along all the lifespan. The possibility to detect dynamic changes in the efficiency of this resilience mechanism along all the lifespan—and not only in the adult or older adults' populations—is, in our opinion, one of the most interesting advantages, not only for the measurement itself but also for evaluating the possible effectiveness of interventions. Another key advantage of the NAR mechanism is that it can be assessed dynamically across the entire lifespan, including early and mid‐adulthood, rather than being inferred retrospectively through static reserve proxies.

Preventive interventions might indeed be pivotal for healthy and successful aging but also rehabilitation in different psychiatric or neurological populations might be valuable. Given the intimate connection between the heart and the brain, several studies have examined the effects of a range of interventions on both brain and heart health (Thayer et al. 2021) and usually findings confirm that they benefit both the brain and the heart. Psychological intervention such as CBT, mindfulness‐based stress reduction (MBSR) intervention, integrative body‐mind training (IBMT) proved good outcomes on emotional and physical health (see Thayer et al. 2021).

Finally, it is worth noting that both bio/neurofeedback and neurostimulation programs seem to be promising. On the one hand preliminary results of bio and neurofeedback trainings that target the previously cited physiological index (mainly HRV) have proved positive effects on executive functioning (Tinello et al. 2022), emotional regulation (Lehrer et al. 2020; Tosti et al. 2024) and relief of depression symptoms (Pizzoli et al. 2021). On the other hand, a meta‐analysis has proved that TMS or transcranial direct current stimulation (TdCS) on prefrontal regions was associated with increased HRV and decreased HR and blood pressure (Makovac et al. 2017; Schmaußer et al. 2022). However, many of these studies have focused their attention only on HRV and not on the CSAB included in the NAR hypothesis, opening again the call for further research needed for a better understanding of the brain‐heart relationship and its complex mechanisms.

## 6 Conclusion

In conclusion, this perspective article proposes and operationalizes NAR as a flexible, dynamic interaction between the autonomic and central nervous systems. By enabling self‐regulation and efficient adaptation to cognitive and emotional stress across the lifespan, NAR emerges as a novel resilience mechanism capable of shaping individual trajectories of cognitive aging and mental health. More in detail, NAR would protect individuals against pro‐inflammatory processes and interact with other mechanisms (e.g., reserve, maintenance, compensation) to help sustain cognitive and mental health in the face of aging and/or adverse brain‐related changes. However, compared to the other proposed mechanisms, NAR would have the advantage of being measurable in a non‐invasive and dynamic manner throughout the entire lifespan. Further studies, better if longitudinal, are needed to verify all these hypotheses and to deepen the interaction between NAR and other forms of resilience such as cognitive and affective reserve. As a theoretical construct, NAR requires empirical validation, particularly through longitudinal and multimodal studies aimed at establishing its predictive specificity and clinical relevance. In the context of a constant population aging, the study of mechanisms such as NAR might be pivotal for the construction of preventive intervention: its focus on the entire lifespan, and its easy and non‐invasive measurement could be of fundamental importance in the field of affective and cognitive neuroscience of aging.

## Author Contributions

**Simona C. S. Caravita:** funding acquisition, writing – review and editing, supervision, validation. **Giulia Fusi:** conceptualization, funding acquisition, writing – original draft, writing – review and editing, visualization. **Barbara Colombo:** writing – review and editing, funding acquisition, conceptualization, supervision, validation.

## Funding

Funded by NextGenerationEU and the Italian Ministry of University and Research (MUR) through the DM 737/2021 (code: YIRGDSUSFG24).

## Conflicts of Interest

The authors declare no conflicts of interest.

## Data Availability

Data sharing not applicable to this article as no datasets were generated or analyzed during the current study.
